# Clinical experience with fluoroquinolones for treatment of severe *Chlamydia psittaci* pneumonia with invasive mechanical ventilation in ICU: a retrospective study

**DOI:** 10.3389/fmed.2025.1682699

**Published:** 2026-01-13

**Authors:** Binbin Feng, Yueliang Chen, Zhenwei Yu, Shuping Song, Jincheng Ruan, Feng Guo

**Affiliations:** 1 Department of Critical Care Medicine, Sir Run Run Shaw Hospital, Zhejiang University School of Medicine, Hangzhou, Zhejiang, China; 2 Department of Pharmacy, Zhejiang University School of Medicine, Sir Run Run Shaw Hospital, Hangzhou, Zhejiang, China; 3 Department of Anus and Intestine Surgery, The Tongde Hospital of Zhejiang Province, Hangzhou, Zhejiang, China

**Keywords:** *Chlamydia psittaci* pneumonia, clinical characteristics, fluoroquinolones, severe pneumonia, treatment

## Abstract

**Background:**

In this study, we sought to investigate the clinical manifestations, laboratory findings and radiographic features of severe *Chlamydia psittaci* pneumonia requiring invasive mechanical ventilation in the intensive care unit (ICU), as well as evaluate the role of fluoroquinolones in its treatment.

**Methods:**

We retrospectively reviewed the clinical data of 14 patients diagnosed with severe *C. psittaci* pneumonia at Zhejiang University School of Medicine, Sir Run Run Shaw Hospital from December 2018 to November 2022. We collected the clinical characteristics, laboratory examination results, imaging features, treatment and prognosis. We used the SPSS 26.0 software to perform statistical analysis.

**Results:**

Most patients presented high fever (≥39 °C), shortness of breath and cough, as well as extrapulmonary symptoms such as fatigue, dizziness, muscle pain and headache. There were also significant increases in white blood cells, C-reactive protein (CRP), procalcitonin (PCT), alanine aminotransferase (ALT), aspartate aminotransferase (AST), lactate dehydrogenase (LDH), and D-dimer, while lymphocytes decreased significantly. Primary chest imaging features included consolidation, exudation and pleural effusion. All 14 patients received fluoroquinolones-based treatment and recovered.

**Conclusion:**

The clinical manifestations of severe *C. psittaci* pneumonia are nonspecific. Fluoroquinolones-based treatment may be effective in severe *C. psittaci* pneumonia with invasive mechanical ventilation in ICU. For severe cases of *C. psittaci* pneumonia, the treatment regimen may require an intravenous fluoroquinolone-based approach.

## 1 Introduction

Psittacosis is an acute infectious disease caused by *Chlamydia psittaci* (*C. psittaci*). *C. psittaci* is a Gram-negative, obligate intracellular bacterium. There are 10 known genotypes based on sequencing the major outer-membrane protein gene, ompA, of *C. psittaci*. Each genotype has overlapping host preferences and virulence characteristics (1). It is estimated that psittacosis accounts for 8.0% of severe CAP patients in China (2). Despite its relatively low fatality rate (1%) (3), the fatality rate may reach to 15–20% for severe psittacosis patients who are not treated timely. This infection mainly results from contact with aerosols of birds’ or poultry’s urine, feces and excrement. Meanwhile, latest studies have shown that human-to-human transmission may occur. The circumstances of human-to-human transmission are unclear, but close contact and infection severity may be risk factors according to the literatures (4, 5). The clinical manifestations range from general symptoms of infection to severe acute respiratory syndrome and systemic diseases. Psittacosis is difficult to diagnose correctly due to limitations of traditional testing methods. The conventional diagnostic methods for psittacosis include culture, serologic testing, and molecular detection. These diagnostic tests are all associated with significant limitations. Serologic testing remains the primary method for confirming psittacosis; however, a fourfold increase in antibody titer or an IgM titer ≥16 is required to establish diagnostic significance. Serologic testing for psittacosis may exhibit cross-reactivity with other members of the Chlamydia genus, potentially compromising its diagnostic specificity. This was particularly observed during the COVID-19 pandemic, when co-infections with other Chlamydia species were frequently identified. During the COVID-19 pandemic, patients co-infected with *Chlamydia pneumoniae* or *Mycoplasma pneumoniae* exhibited worse respiratory or radiographic features and higher rates of intensive care unit admission compared with those infected with SARS-CoV-2 alone (6). Therefore, clinicians often ignore them and miss the best treatment time (7). Metagenomic next-generation sequencing (mNGS) has enhanced the detection rate of psittacosis pneumonia (8). However, at present, most physicians have little understanding of *C. psittaci*, especially for severe patients, and lack experience in diagnosis and treatment.

Tetracyclines are preferred for the first-line treatment of *C. psittaci* pneumonia, while fluoroquinolones and macrolides have been recommended as alternatives (9). The oral tetracyclines formulations are not suitable for critically ill patients due to gastrointestinal side effects, hepatotoxicity and limited availability of intravenous formulations in hospitals. The use of antibiotics in the poultry and pet bird industries may have contributed to the drug resistance of *C. psittaci* (5, 10).

The 2019 IDSA/ATS CAP guidelines recommended quinolone or quinolone in combination with β-lactam for inpatient adults with severe CAP (11). In clinical practice, fluoroquinolones are the most commonly used empiric antibiotics for severe psittacosis pneumonia. Fluoroquinolones are effective against *Chlamydia psittaci in vitro*. Despite these in vitro data, there are few clinical studies evaluating the role of fluoroquinolones in psittacosis, and few clinicians would recommend their use as first-line agents (12). Thus, the objective of this study was to describe the clinical characteristics of psittacosis pneumonia and review fluoroquinolones for the treatment of severe *C. psittaci* pneumonia with invasive mechanical ventilation in ICU.

In this study, we identified patients with severe *C. psittaci* pneumonia, confirmed by metagenomic next-generation sequencing (mNGS), who required invasive mechanical ventilation in the intensive care unit (ICU) between December 2018 and November 2022. We retrospectively summarized the characteristics and treatment process for these patients to remind physicians that psittaci pneumonia and empiric fluoroquinolones-based treatment for CAP should also be considered.

## 2 Methods

### 2.1 Study design

Clinical data of 14 patients with severe pneumonia caused by *C. psittaci* admitted to Zhejiang University School of Medicine Sir Run Run Shaw Hospital ICU, between December 2018 and November 2022, were retrospectively analyzed in this study. All patients had mNGS because of their critical condition or unsatisfactory empirical treatment effect. All the cases were diagnosed by mNGS combined with clinical manifestations and other laboratory diagnostic methods. Clinical data of each patient were obtained from the electronic medical record system, including the basic information, whether there was any poultry contact history, concurrent primary diseases, clinical manifestations, laboratory tests, mNGS results, imaging manifestations, antibiotic use, complications and disease outcomes. The current study was approved by the hospital ethics committee (Approval No. 2025-2240-01), and all data were anonymized before analysis.

### 2.2 Inclusion criteria

Patients diagnosed with *C. psittaci* pneumonia should meet the following three diagnostic criteria: ① Meet the diagnostic criteria for severe community-acquired pneumonia (11) and receive invasive mechanical ventilation; ② *C. psittaci* was detected by mNGS in alveolar lavage fluid (BALF) or blood; ③ Routine pathogenic tests in the hospital, including blood culture, sputum culture and alveolar lavage fluid, were negative, and no other pathogens were detected. Respiratory failure was defined as an oxygenation index <300 mmHg (1 mmHg = 0.133 kPa), and shock was defined as needing fluid resuscitation and drugs to maintain blood pressure.

### 2.3 Exclusion criteria

① Cases with incomplete medical records. ② Cases where mNGS indicates the presence of other major pathogenic bacteria.

### 2.4 Laboratory and imaging examination

The total number of white blood cells, the proportion of neutrophils, lymphocytes, C-reactive protein (CRP), procalcitonin (PCT), alanine aminotransferase (ALT), aspartate aminotransferase (AST), serum creatinine (SCr), blood urea nitrogen (BUN), lactate dehydrogenase (LDH), creatine kinase (CK), D-dimer and the oxygenation index on the day of admission to the ICU were recorded.

The chest CT of all patients was completed before ICU admission, and the distribution and type of pulmonary lesions were described. The patient’s alveolar lavage fluid or blood was obtained and sent to mNGS for testing. The mNGS was performed by a professional, commercially available testing service. mNGS took 24–48 h from sample receipt to final report (13).

### 2.5 Treatment and outcome

Serial data on antibiotic utilization and associated laboratory trends were prospectively documented. Record the clinical cure rate, the 30 day mortality, the length of ICU stay, the duration of fever withdrawal, and drug-related adverse events.

### 2.6 Statistical analysis

Data processing and statistical analysis using SPSS 26.0 statistical analysis package. Categorical data were summarized using frequencies and percentages. Continuous data were summarized using median and interquartile range (IQR).

## 3 Results

### 3.1 Patient baseline characteristics

We enrolled 14 patients (78.6% male; male:female ratio 3.67:1), with a median age of 63 (55–67) years. Most patients (64.3%) had underlying medical conditions. Among the 14 patients, 4 cases had contact with domestic poultry before the onset of the disease.

Of the 14 cases, 8 (57.1%) occurred in autumn and winter, with a pronounced peak incidence in November and December. The time from symptom onset to hospital admission was 6.5 (5–8.5) days. Among the 14 patients, 13 (92.8%) presented with fever, with a mean peak temperature was 39.6 (39.2–40.0) °C. Common accompanying symptoms included shortness of breath and cough (14/14, 100%), fatigue (10/14, 71.4%), dizziness (4/14, 28.6%), muscle pain (2/14, 14.3%) and headache (1/14, 7.1%). All patients (100%) presented with respiratory failure at ICU admission and subsequently required endotracheal intubation with mechanical ventilation. Demographic characteristics of the patients are shown in Table 1.

**Table 1:** Demographic characteristics of the included patients.

Variable	Number of cases (%)	Median (IQR)
Sex, *n* (%)		
Male	11 (78.6%)	
Female	3 (21.4%)	
Age (years)		63 (55–67)
Comorbidities, *n* (%)		
Hypertension	5 (35.7%)	
Diabetes	2 (14.3%)	
Psychiatric disorders	2 (14.3%)	
Neoplastic disease	1 (7.1%)	
Atherosclerosis	1 (7.1%)	
Gastrointestinal bleeding	1 (7.1%)	
Permanent pacemaker implantation	1 (7.1%)	
Clinical features		
Fever >38.5 °C	13 (92.8%)	39.6 (39.2–40.0)
Shortness of breath, cough	14 (100%)	—
Muscle pain	2 (14.3%)	—
Fatigue	10 (71.4%)	—
Headache	1 (7.1%)	—
Dizziness	4 (28.6%)	—
Shock	3 (21.4%)	
SOFA score		7 (5–12)
APACHE II score		17 (16–24)
Laboratory data		
Elevated WBC (×10^9^/L)	64.3% (9/14)	10.9 (6.8, 13.3)
Elevated percentage of neutrophils	100% (14/14)	96 (93, 98)
Elevated lymphocyte (×10^9^/L)	100% (14/14)	0.3 (0.2, 0.5)
Elevated CRP (μg/L)	100% (14/14)	343 (188, 388)
Elevated PCT (ng/mL)	100% (14/14)	3.4 (1.0, 13.2)
Elevated SCr (μmol/L)	35.7% (5/14)	91 (54, 187)
Elevated BUN (mmol/L)	64.3% (9/14)	9.3 (7.6, 13.9)
Elevated ALT (U/L)	78.6% (11/14)	68 (50, 139)
Elevated AST (U/L)	92.8% (13/14)	111 (79, 282)
Elevated LDH (IU/L)	100% (14/14)	570 (492, 841)
Elevated CK (IU/L)	64.3% (9/14)	437 (176, 3,642)
Elevated D-dimer	78.6% (11/14)	5.5 (2.9–7.4)
Declined P/F	100% (14/14)	143 (107, 194)

SOFA, Sequential organ failure assessment; APACHE II, Acute physiology and chronic health evaluation II; WBC, White blood cells; CRP, C-reactive protein; PCT, Procalcitonin; SCr, Serum creatinine; BUN, Blood urea nitrogen; ALT, Alanine aminotransferase; AST, Aspartate aminotransferase; LDH, Lactate dehydrogenase; CK, Creatine kinase; P/F, PaO₂/FiO₂ ratio.

### 3.2 Imaging characteristics

All patients completed chest CT examinations before ICU admission. The most common findings were pulmonary infiltrates and consolidation with air-containing bronchial shadow, observed in 14 cases (14/14, 100%). On chest computed tomography (CT), bilateral pulmonary involvement was observed in 10 cases (71.4%), with unilateral involvement in 4 cases (28.6%)-affecting the left lung in 1 case and the right lung in 3 cases.

### 3.3 Treatment and prognosis

All patients received fluoroquinolones therapy after the diagnosis of *C. psittaci* by mNGS (Table 2). Most patients received initial antimicrobial treatment with carbapenem, either alone or in combination with an anti-MRSA agent as the empiric therapy. Among them, 6 cases (42.9%) did not cover the identified pathogens in initial empiric treatment. Before diagnosis, 4 cases were empirically treated with moxifloxacin, 1 case was treated with levofloxacin, and 3 cases were treated with azithromycin. After confirmation of the diagnosis, the therapeutic approach was adjusted as follows: 4 cases (28.6%) received moxifloxacin; 2 cases (14.3%) received a combination therapy with moxifloxacin plus azithromycin; 1 case (7.14%) received a combination of moxifloxacin and tigecyclines; 7 cases (50%) received a combination of quinolones and doxycycline. Fluoroquinolones were administered intravenously with moxifloxacin at a dose of 400 mg once daily, and levofloxacin at a dose of 500 mg once daily. Doxycycline was administered via nasogastric tube at a dose of 100 mg every 12 h. Transient hepatic transaminase elevations were observed in 3 cases (2 possibly moxifloxacin-induced, 1 doxycycline-related). The course of the target treatment was 21.0 days. The duration of fever withdrawal was approximately 2.5 (1–6) days, accompanied by a significant decrease in CRP. Seven cases were treated with methylprednisolone (40–60 mg, bid).

**Table 2:** Treatment data of patients with *Chlamydia psittaci* pneumonia.

Treatment	All (*N* = 14)	Median (IQR)
Fluoroquinolones	4 (28.6%)	
Fluoroquinolone + azithromycin	2 (14.33%)	
Fluoroquinolone + tigecyline	1 (7.14%)	
Fluoroquinolone + doxycycline	7 (50%)	
Invasive ventilation	14 (100%)	
CRRT	4 (28.6%)	
ECMO	2 (14.3%)	
Outcome		
Days of ICU stay		14 (12–17)
Survival	14 (100%)	

ECMO, Extracorporeal membrane oxygenation; CRRT, Continuous renal replacement therapy.

During the treatment course, 2 cases (14.3%) required extracorporeal membrane oxygenation (ECMO) support. Four cases (28.6%) underwent continuous renal replacement therapy (CRRT). At discharge, 3 cases (21.4%) renal function recovered, while 1 case (7.1%) required ongoing hemodialysis. The length of ICU stay was 14 (12–17) days. Both fluoroquinolones monotherapy and combination therapy regimens were associated with favorable clinical outcomes. Detailed information about the included patients is shown in Table 3.

**Table 3:** Detailed information about the included patients.

Case#	Age (years)/Sex	Underlying diseases	Poultry exposure	Onset time (days)	Symptoms	Signs	Inflammatory biomarkers	Biochemistry	Imaging	Antibiotics before diagnosis	Antibiotics after diagnosis	Fever time (d)	ICU stays (d)
1	57/Female	None	Indirect	8	High-fever Shortness of breath, cough	40.0 °C Shock	WBC: 5100/μL NE: 4500/μL LY: 490/μL CRP: 104.6 mg/L PCT: 1.04 ng/mL	SCr: 47 μmol/L ALT: 87 U/L D-dimer: 4.3 μg/mL	Consolidation with air-containing bronchial shadow, pleural effusion	Meropenem Vancomycin	Levofloxacin + doxycycline	10	22
2	67/Male	Neoplastic disease	Indirect	4	Fever Shortness of breath, cough Muscle pain, Fatigue	38.7 °C Moist rales	WBC: 12100/μL NE: 11900/μL LY: 120/μL CRP: 499.0 mg/L PCT: 25.4 ng/mL	SCr: 91 μmol/L ALT: 33 U/L D-dimer: 3.1 μg/mL	Consolidation with air-containing bronchial shadow, pleural effusion	Imipenem/cilastatin + moxifloxacin	Levofloxacin + doxycycline	7	52
3	55/Male	Psychiatric disorders	Indirect	7	High-fever Shortness of breath, cough	39.6 °C Moist rales	WBC: 29300/μL NE: 28500/μL LY: 350/μL CRP: 383.3 mg/L PCT:0.42 ng/mL	SCr: 56 μmol/L ALT: 138 U/L D-dimer: 1.2 μg/mL	Consolidation with air-containing bronchial shadow, pleural effusion, pleural effusion	Imipenem/cilastatin + azithromycin	Levofloxacin + doxycycline	10	14
4	69/Female	None	Indirect	10	High-fever Shortness of breath, cough Fatigue	39.2 °C Moist rales	WBC: 13100/μL NE: 12800/μL LY: 100/μL CRP: 111.4 mg/L PCT: 0.26 ng/mL	SCr: 30 μmol/L ALT: 57 U/L D-dimer: μg/mL	Consolidation with air-containing bronchial shadow, pleural effusion	Imipenem/cilastatin + vancomycin	Moxifloxacin	1	18
5	68/Male	Psychiatric disorders	Indirect	5	High-fever Shortness of breath, cough Fatigue	40.1 °C Moist rales	WBC: 6400/μL NE: 6200/μL LY: 200/μL CRP: 410.4 mg/L PCT: 5.64 ng/mL	SCr: 105 μmol/L ALT: 40 U/L D-dimer: 7.2 μg/mL	Consolidation with air-containing bronchial shadow	Piperacillin/tazobactam + moxifloxacin	Moxifloxacin + doxycycline	2	14
6	66/Male	Hypertension Atherosclerosis Permanent pacemaker implantation	Poultry	5	Dizziness Fatigue Shortness of breath, cough	None	WBC: 12400/μL NE: 11700/μL LY: 280/μL CRP: 323.9 mg/L PCT: 34.2 ng/mL	SCr: 558 μmol/L ALT:313 U/L D-dimer: 8.8 μg/mL	Consolidation with air-containing bronchial shadow	Imipenem/cilastatin + azithromycin	Moxifloxacin + doxycycline	2	11
7	54/Female	None	Poultry	10	High-fever Shortness of breath, cough Headache	40.6 °C Moist rales Shock	WBC: 14000/μL NE: 13600/μL LY: 200/μL CRP: 350.6 mg/L PCT: 3.3 ng/mL	SCr: 45 μmol/L ALT: 31 U/L D-dimer: 6.6 μg/mL	Consolidation with air-containing bronchial shadow	Meropenem + linezolid	Moxifloxacin	2	14
8	73/Male	Hypertension Diabetes	Poultry	4	High-fever Shortness of breath, cough Fatigue	39.9 °C Moist rales	WBC: 10600/μL NE: 9600/μL LY: 610/μL CRP: 198.9 mg/L PCT: 1.0 ng/mL	SCr: 88 μmol/L ALT: 68 U/L D-dimer: 2.7 μg/mL	Consolidation with air-containing bronchial shadow	Meropenem + moxifloxacin	Moxifloxacin	6	11
9	51/Male	Hypertension	Indirect	7	High-fever Shortness of breath, cough Dizziness	39.1 °C Moist rales	WBC: 11200/μL NE: 10900/μL LY: 210/μL CRP: 206.4 mg/L PCT: 7.2 ng/mL	SCr: 422 μmol/L ALT: 53 U/L D-dimer: 8.3 μg/mL	Consolidation with air-containing bronchial shadow	Meropenem + moxifloxacin	Moxifloxacin + doxycycline	6	14
10	59/Male	Hypertension Diabetes	Indirect	5	High-fever Shortness of breath, cough Fatigue	39.5 °C Shock	WBC: 14500/μL NE: 13900/μL LY: 450/μL CRP: 348.9 mg/L PCT: 2.8 ng/mL	SCr: 112 μmol/L ALT: 64 U/L D-dimer: 2.4 μg/mL	Consolidation with air-containing bronchial shadow, pleural effusion	Meropenem + teicoplanin	Moxifloxacin + azithromycin	3	12
11	53/Male	None	Indirect	5	High-fever Shortness of breath, cough Fatigue	39.2 °C	WBC: 6300/μL NE: 5900/μL LY: 220/μL CRP: 343.9 mg/L PCT: 17.6 ng/mL	SCr: 396 μmol/L ALT: 315 U/L D-dimer: 5.5 μg/mL	Consolidation with air-containing bronchial shadow, pleural effusion	Meropenem	Moxifloxacin + azithromycin	1	17
12	66/Male	None	Indirect	10	High-fever Shortness of breath, cough Fatigue Dizziness	39.6 °C Moist rales	WBC: 10400/μL NE: 9900/μL LY: 260/μL CRP: 403.5 mg/L PCT: 3.6 ng/mL	SCr: 66 μmol/L ALT: 67 U/L D-dimer: 4.1 μg/mL	Consolidation with air-containing bronchial shadow, pleural effusion	Piperacillin/tazobactam + azithromycin	Moxifloxacin + doxycycline	1	16
13	67/Male	Gastrointestinal bleeding	Indirect	7	High-fever Shortness of breath, cough Fatigue Dizziness, muscle pain	40.0 °C Dry and moist rales	WBC: 6900/μL NE: 6500/μL LY: 230/μL CRP: 343.2 mg/L PCT: 11.7 ng/mL	SCr: 90 μmol/L ALT: 142 U/L D-dimer: 7.6 μg/mL	Consolidation with air-containing bronchial shadow, pleural effusion	Imipenem/cilastatin + levofloxacin	Moxifloxacin	1	10
14	56/Male	Hypertension	Poultry	6	High-fever Shortness of breath, cough Fatigue	39.7 °C Moist rales	WBC: 7100/μL NE: 6500/μL LY: 520/μL CRP: 156.6 mg/L PCT: 2.6 ng/mL	SCr: 117 μmol/L ALT: 79 U/L D-dimer: 6.7 μg/mL	Consolidation with air-containing bronchial shadow	Cefoperazone/sulbactam	Moxifloxacin + tigecycline	3	14

## 4 Discussion

We performed a retrospective analysis of fluoroquinolones for treating severe *C. psittaci* pneumonia. These results demonstrate that fluoroquinolones-based treatment was highly successful in severe *C. psittaci* pneumonia with invasive mechanical ventilation in ICU.

The intracellular activity of quinolones against *C. psittaci* showed activity against *C. psittaci in vitro* (14–16). The efficacy of fluoroquinolones in the treatment of severe psittacosis pneumonia remains controversial. Moreover, few studies have evaluated the efficacy of quinolone combination therapy compared to monotherapy.

Previous studies showed that patients with psittacosis had a good prognosis after a combination of quinolones and doxycycline. Zhang et al. (17) observed that concomitant treatment of quinolones and doxycycline was effective in 8/14 (57.1%) cases in severe patients. Yin et al. (8) found that combination therapy with moxifloxacin and doxycycline demonstrated efficacy in 13/32 severe cases. Tang et al. (18) reported there were no significant differences between patients treated with fluoroquinolones initially and fluoroquinolones combined with tetracycline in severe patients accompanied by acute hypoxic respiratory failure.

Several reports have described the treatment of *C. psittaci* pneumonia with quinolone monotherapy. Yang et al. (19) observed usage of quinolones in 116 patients was associated with shorter length of hospital stay and fever duration; patients may benefit from early treatment with quinolone. Yang et al. (20) reported that quinolone was effective in 17/27 (62.9%) treated severe patients.

In this study, only four cases were treated with fluoroquinolones until recovery, whereas the remaining patients were quickly switched to quinolone-based treatment after the diagnosis of psittacosis. All cases showed good efficacy and quick recovery. These results indicate that fluoroquinolones are effective in the treatment of *C. psittaci*, and fluoroquinolones could be used for severe *C. psittaci* pneumonia. Fluoroquinolones could be used for severe *C. psittaci* pneumonia as monotherapy or combination therapy with tetracyclines or macrolides. This allows for a seamless transition from intravenous to oral therapy (sequential therapy) following clinical stabilization, thereby facilitating the completion of the full treatment course.

According to the research report, *C. psittaci* pneumonia mainly occurs in middle-aged and older adults (45–70 years old) (21). In this study, 78.6% cases with *C. psittaci* infection are male, and 85.7% cases are 55 years old or older. Exposure to birds or poultry is a significant risk factor for psittacosis. Therefore, patients with community-acquired pneumonia who have a history of contact with birds or domestic birds should be alert to *C. psittaci* infection. But in our study, only 28.6% of the cases reported contact. When patients with community-acquired pneumonia have a critical illness, poor treatment effect, and particular suspicious pathogens, the corresponding mNGS detection can improve the diagnostic rate, avoid further aggravation and deterioration of the disease, and shorten the hospitalization time of patients.

The clinical manifestations of *C. psittaci* pneumonia are high fever, shortness of breath, cough, fatigue, dizziness, muscle pain, headache, etc. The clinical manifestations vary greatly and may be asymptomatic for a long time or mainly manifested by lung involvement, sometimes only showing a fever of unknown origin and lack of respiratory symptoms (22).

Chest CT shows invasive lung lesions, which can affect multiple organs in severe cases. In this study, patients mainly had lesions in the bilateral, middle-lower lobe, and multilobar disease, generally presented as consolidation. In patients with *C. psittaci* pneumonia, the extent of the lesion is closely related to the severity of the disease, and the risk of respiratory failure with multilobar lesions is higher than in other types of CAP. Further, 71.4% of the patients had pleural effusions, which was higher than the incidence rates of 15% reported in Australia (23) and 44.4% reported in another Chinese study (24). Concomitant pleural effusion can be considered related to the involvement of the pleural membrane and secondary hypoproteinemia.

We found that lymphocytopenia occurred in 93% of critically ill patients. Lymphocytopenia is a significant finding of patients with severe community-acquired infections. Lymphopenia was independently associated with the risk of ICU admission and higher in-hospital and 30 day mortality in patients with CAP and sepsis (25). Therefore, it is critical to identify lymphopenia in hospitalized patients with CAP and sepsis, as lymphopenia might serve to prioritize patients with CAP and sepsis who require or will shortly require critical care.

In this study, the neutrophil percentage, CRP, PCT, D-dimer, AST, lactate dehydrogenase and CK were significantly increased. The clinical utility of D-dimer measurement in patients with CAP is still unclear. In this study, D-dimer levels were slightly elevated, as reported by Cerda-Mancillas et al. (26). Intra and extravascular activation of coagulation along acute inflammation is thought to be associated with acute and chronic lung damage in CAP by fibrin deposition in alveoli and lung interstitium. D-dimer plasma levels are associated with the severity of CAP (26).

Considering severe pneumonia and severe systemic inflammatory reaction, glucocorticoids were used in 13 cases (92.8%) with higher body temperatures, showing good clinical results without any complications. SCCM’s revised guidelines support corticosteroid therapy in patients with severe CAP and ARDS for its probable role in reducing hospital mortality and the requirement for invasive mechanical ventilation, albeit with an increased risk for hyperglycemia (27).

In conclusion, the manifestations of *C. psittaci* pneumonia are diverse and with unique features in clinical symptoms, laboratory tests, and chest imaging. The overall prognosis is good, but severe patients need CRRT and ECMO support. Appropriate treatment can significantly shorten the course of the disease and improve the prognosis. For patients with suspected *C. psittaci* pneumonia, a fluoroquinolone-based regimen may be considered as part of the initial empirical therapeutic strategy. Especially patients who have a history of contact with birds or domestic birds, lymphocytopenia, and high fever. Fluoroquinolones could be used for severe *C. psittaci* pneumonia as monotherapy or combination therapy with tetracyclines or macrolides.

However, this study has some limitations. Firstly, it was a single-center study conducted in a tertiary-care teaching hospital, which may introduce bias. Secondly, it was a retrospective study, and the sample size was limited. Lastly, the retrospective study may not fully represent the clinical features and treatment of *Chlamydia psittaci* pneumonia. Future prospective studies with larger, multicenter samples should be conducted. Despite this, the results suggest fluoroquinolones therapy is effective in patients with severe psittacosis pneumonia.

## Data Availability

The raw data supporting the conclusions of this article will be made available by the authors, without undue reservation.
